# Limitations to current methods to estimate cause of death: a validation study of a verbal autopsy model

**DOI:** 10.12688/gatesopenres.13132.3

**Published:** 2021-05-05

**Authors:** Clara Menéndez, Llorenç Quintó, Paola Castillo, Carla Carrilho, Mamudo R. Ismail, Cesaltina Lorenzoni, Fabiola Fernandes, Juan Carlos Hurtado, Natalia Rakislova, Khátia Munguambe, Maria Maixenchs, Eusebio Macete, Inacio Mandomando, Miguel J Martínez, Quique Bassat, Pedro L Alonso, Jaume Ordi

**Affiliations:** 1 Barcelona Institute for Global Health (ISGlobal), Hospital Clinic of Barcelona, Universitat de Barcelona, Barcelona, Spain; 2 Centro de Investigação em Saúde de Manhiça, Maputo, Mozambique; 3 Consorcio de Investigación Biomédica en Red de Epidemiología y Salud Pública (CIBERESP), Madrid, Spain; 4 Pathology, Hospital Clinic of Barcelona, Universitat de Barcelona, Barcelona, Spain; 5 Pathology, Maputo Central Hospital, Maputo, Mozambique; 6 Faculty of Medicine, Eduardo Mondlane University, Maputo, Mozambique; 7 Microbiology, Hospital Clinic of Barcelona, Universitat de Barcelona, Barcelona, Spain; 8 ICREA, Catalan Institution for Research and Advanced Studies, Barcelona, Spain

**Keywords:** Validation, verbal autopsy, cause of death, complete diagnostic autopsy, Mozambique

## Abstract

**Background**: Accurate information on causes of death (CoD) is essential to estimate burden of disease, track global progress, prioritize cost-effective interventions, and inform policies to reduce mortality. In low-income settings, where a significant proportion of deaths take place at home or in poorly-resourced peripheral health facilities, data on CoD often relies on verbal autopsies (VAs). Validations of VAs have been performed against clinical diagnosis, but never before against an acceptable gold standard: the complete diagnostic autopsy (CDA).

**Methods: **We have validated a computer-coded verbal autopsy method –the InterVA- using individual and population metrics to determine CoD against the CDA, in 316 deceased patients of different age groups who died in a tertiary-level hospital in Maputo, Mozambique between 2013 and 2015.
* *

**Results: **We found a low agreement of the model across all age groups at the individual (kappa statistic ranging from -0.030 to 0.232, lowest in stillbirths and highest in adults) and population levels (chance-corrected cause-specific mortality fraction accuracy ranging from -1.00 to 0.62, lowest in stillbirths, highest in children). The sensitivity in identifying infectious diseases was low (0% for tuberculosis, diarrhea, and disseminated infections, 32% for HIV-related infections, 33% for malaria and 36% for pneumonia). Of maternal deaths, 26 were assigned to eclampsia but only four patients actually died of eclampsia.

**Conclusions: **These findings do not lead to building confidence in current estimates of CoD. They also call to the need to implement autopsy methods where they may be feasible, and to improve the quality and performance of current VA techniques.

## Introduction

Global and disease-specific health statistics are regularly published and constitute an essential tool to define priorities and goals, identify inequalities, and track progress, including the achievement of global targets such as the health-related Sustainable Development Goals (SDGs). Insufficient confidence in the accuracy of estimates, particularly in those related to cause of death (CoD) has been indicated as a constraint to reduce mortality globally ^1, 2^. This lack of precise information on CoD in many low-income settings is largely explained by the significant number of deaths that occur either at home or at poorly resourced health facilities, with significant limitations of both qualified personnel as well as accurate diagnostic methods; but also to the very limited number of diagnostic autopsies performed partly due to the massive shortfall of trained pathologists ^1, 2^. As such, CoD in low-income settings, continues to rely on estimates based on clinical records and verbal autopsies (VAs).

Clinical errors, which are common even in well-equipped hospitals, are more frequent in resource-restricted settings ^3– 5^. On the other hand, VAs remain the most practical and commonly used approach to estimate CoD at the population level in low-income settings ^6^. A verbal autopsy consists of a structured interview to witnesses of the death subsequently interpreted and coded by physicians or using computerized methods. The method has shown to provide inconsistent results over time and place ^7^. In addition, its diagnostic accuracy depends on the CoD, being high when the disease has a characteristic and well defined set of signs and symptoms, but much lower for conditions with unspecific symptoms, notably, malaria and acute respiratory infection in children, or meningitis in all age groups ^6^. This results in frequent misclassifications of the CoD, which in turn leads to inaccurate cause-specific mortality rates ^6^. Computerized methods of interpretation of the VA questionnaire have been developed to overcome some of the limitations of the VA technique. These methods are based either on algorithms derived from deaths with a medically confirmed CoD, or on probabilistic analyses ^8^.

Computerized VA methods have been validated against physician-certified VA and clinical records ^9– 11^. However, neither computer-coded VA, nor physician-certified VA techniques have been validated against the complete diagnostic autopsy (CDA), the true gold standard for CoD determination. We present herein the results of a validation study of a commonly used computer-coded VA method, the InterVA (Interpreting Verbal Autopsy) model against the CDA in a series of deaths occurring in Maputo, Mozambique.

## Methods

### Study design and setting

The study included 316 CDA performed to patients who died between 2013 and 2015 at the Maputo Central Hospital, a 1500-bed institution that serves as the referral center for other hospitals in Mozambique. All the patients included in this analysis fulfilled the following criteria: (1) a CDA requested by the clinician as part of the medical evaluation of the patient and (2) informed consent to perform the autopsy given by the relatives. The following exclusion criterion was established: death of traumatic origin. In order to select only two cases per day from among the daily CDA requests received at the department of pathology (between 5 and 12 per day) without introducing selection biases, the two patients with death recorded before and closest to the time of 8:00 A.M. were included in the study. All maternal deaths that occurred in the study period were included.

From the 316 cases, 18 (6%) were stillbirths, 41 (13%) were neonates, 54 (17%) were children 1 month-15 years of age, 91 (29%) were maternal deaths and 112 (35%) were other adults. Written informed consent to perform the autopsy was obtained from the relatives of the deceased patients. In Maputo province malaria transmission is reported to be low (3%) and HIV prevalence is high (22%) ^12, 13^.

This study received approval by the Clinical Research Ethics Committee of the Hospital Clinic of Barcelona, Spain (File 2013/8677) and the National Bioethics Committee of Mozambique (Ref. 342/CNBS/13).

### Determination of the cause of death by the complete diagnostic autopsy

The methodology for CoD determination by the CDA has been described in detail elsewhere ^14– 18^. Briefly, a panel of experts evaluated the CDA macroscopic, microscopic and microbiologic data, as well as the clinical information and assign the CoD. All morbid conditions directly leading to death, any underlying and any other significant conditions possibly contributing to death were codified according to the international classification of diseases, tenth revision (ICD-10, ICD-10 MM for maternal deaths) ^19^. When more than one severe diagnosis was identified, the disease most likely causing the death was considered the final diagnosis ^14– 17^.

### Cause of death assignment by the Verbal Autopsy model

We used the InterVA probabilistic model because it is one of the most commonly implemented VA tools ^20^ and has shown a generally good level of agreement with the physician-coded verbal autopsy approach; it has also the advantage of being a completely reproducible method, reliable and standardized to interpretation ^21, 22^ reducing subjectivity. The InterVA method is based on the Bayes’ theorem and calculates the probability of a set of CoD given the presence of indicators reported in VA interviews ^23, 24^. We used version 4.04 of the model (InterVA-4) since the most recent version (InterVA-5) had not been released yet. In this analysis, the information feeding the model was extracted by the attending physician at the hospital from the clinical record of the deceased individual and from the obstetric record in perinatal deaths (Extended data ^25^: Clinical and epidemiological data collection questionnaire), unified into the WHO 2012 VA standard format ^7^, converted into the 245 input indicators of the VA model, and processed with malaria prevalence set to “low”, and HIV prevalence set to “high” using the InterVA4 package version 1.7.5 implemented in R version 3.5.0 software ^26^. Of the 245 input indicators of the model, 43 could not be extracted from the medical records; 24 (56%) of them were secondary questions, which are not pertinent if certain events did not occur.

### Validation of the model

To validate the VA model across a variety of CoD distributions, 500 cause compositions based on uninformative Dirichlet sampling were generated for each study group ^27^. The performance of the model at the individual level was estimated comparing the CoD established by the CDA with the most probable CoD provided by the model. The Kappa statistic and the chance corrected concordance (CCC) were used as measures of the overall performance of the model (Extended data ^25^: Table S1 and Figure S1) ^28– 30^.

At the population level, cause-specific mortality fractions (CSMFs) were calculated for each CoD and method within each study group. Since the model estimates up to three CoD with associated likelihoods for each cause, all identified CoD were considered as proportional to their partial likelihoods in the rate calculations for the model. In contrast, only one CoD was considered for the CDA and consequently, the associated likelihood was assumed to be 1. The CSMF accuracy (CSMFA) and the chance-corrected CSMFA (CCCSMFA) were calculated to compare the CSMFs determined by the InterVA model with those determined by the CDA (Extended data ^25^: Table S1 and Figure S1) ^28^. All analyses were done in Stata version 15 (Stata Corp., College Station, TX, USA) and R version 3.5.0 (R Core Team, 2017) statistical packages.

## Results

The VA model assigned one CoD in 267 (84%) cases and two CoD in 33 (10%) cases. In 16 (5%) cases the model resulted in a non-conclusive diagnosis. The average likelihood of the model in estimating the first CoD was 90% (range 89% to 99%), and for the second CoD it was 38% (range 35% to 46%) (Extended data ^25^: Table S2). Three of the 316 cases (1%) had a non-conclusive diagnosis in the CDA.

### Assignment of the CoD at the individual level compared with the CDA

In 168/316 cases (53%) the two methods agreed in the CoD. Most of the agreement was in the first CoD, while only in 8 cases the agreement was in the second CoD with a mean likelihood of 38% [95%CI: (33–43)] (Extended data ^25^: Table S3). In 148/316 cases (47%), there was no agreement in the CoD between the two methods.

Overall, the performance of the VA method in assigning a CoD to individual deaths was low ( Table 1). In stillbirths, the sensitivity of the model in identifying infections, fetal growth restriction, and intrapartum and intrauterine hypoxia was 0%. In neonates, the sensitivity was 93% for infectious CoD, while it was 0% and 25% for preterm complications and congenital malformations, respectively. In children, the sensitivity of the model in identifying an infectious disease as CoD was 83%, while it was 0% for the congenital malformations, tumors and other diseases. The sensitivity of the model in identifying maternal mortality causes was low for all conditions except for eclampsia (75%) and obstetric hemorrhage (75%). In other adults, the sensitivity of the model was highest for infectious diseases (68%) and lowest for malignant neoplasms (19%).

**Table 1.** Performance of the InterVA (Interpreting Verbal Autopsy) model compared to the complete diagnostic autopsy at individual-level prediction by study group and cause of death.

Cause of death (CDA)		n	Classification of cases				Sensitivity (%)	Specificity (%)	PPV (%)	NPV (%)
			TP	TN	FP	FN				
**Stillbirths** **(N=18)**	Infections	4	0	14	0	4	0	100	*N/A*	78
	Fetal growth restriction	7	0	10	1	7	0	91	0	59
	Intrapartum hypoxia	3	0	15	0	3	0	100	*N/A*	83
	Intrauterine hypoxia	2	0	16	0	2	0	100	*N/A*	89
	Congenital malformations	0	0	11	7	0	*N/A*	61	0	100
	Non-conclusive	2	1	7	9	1	50	44	10	88
**Neonates** **(N=41)**	Infections	27	25	2	12	2	93	14	68	50
	Congenital malformations	4	1	37	0	3	25	100	100	92
	Preterm complications	5	0	36	0	5	0	100	*N/A*	88
	Intrapartum complication	3	1	36	2	2	33	95	33	95
	Other diseases	2	0	39	0	2	0	100	*N/A*	95
	Non-conclusive	0	0	41	0	0	*N/A*	100	*N/A*	100
**Children** **(N=54)**	Infections	42	35	3	9	7	83	25	80	30
	Congenital malformations	2	0	52	0	2	0	100	*N/A*	96
	Malignant neoplasms	7	0	47	0	7	0	100	*N/A*	87
	Other diseases	3	0	44	7	3	0	86	0	94
	Non-conclusive	0	0	51	3	0	*N/A*	94	0	100
**Maternal** **deaths** **(N=91)**	Infections *	39	11	39	13	28	28	75	46	58
	Abortion	9	0	82	0	9	0	100	N/A	90
	Eclampsia	4	3	64	23	1	75	74	12	98
	Obstetric hemorrhage	16	12	59	16	4	75	79	43	94
	Other obstetric complications	6	0	85	0	6	0	100	*N/A*	93
	Non-obstetric diseases **	16	4	67	8	12	25	89	33	85
	Non-conclusive	1	0	89	1	1	0	99	0	99
**Other** **adults** **(N=112)**	Infections	80	54	20	12	26	68	62	82	43
	Malignant neoplasms	16	3	95	1	13	19	99	75	88
	Other diseases	16	10	66	30	6	62	69	25	92
	Non-conclusive	0	0	110	2	0	*N/A*	98	0	100

**CDA:** complete diagnostic autopsy; **n**: number of cases; **TP:** true positives; **TN:** true negatives; **FP:** false positives; **FN:** false negatives; **PPV:** positive predictive value; **NPV:** negative predictive value; **N/A:** not applicable.

* Includes all infections, both obstetric and non-obstetric.

** Non-obstetric diseases do not include infections.

**Figure 1.**
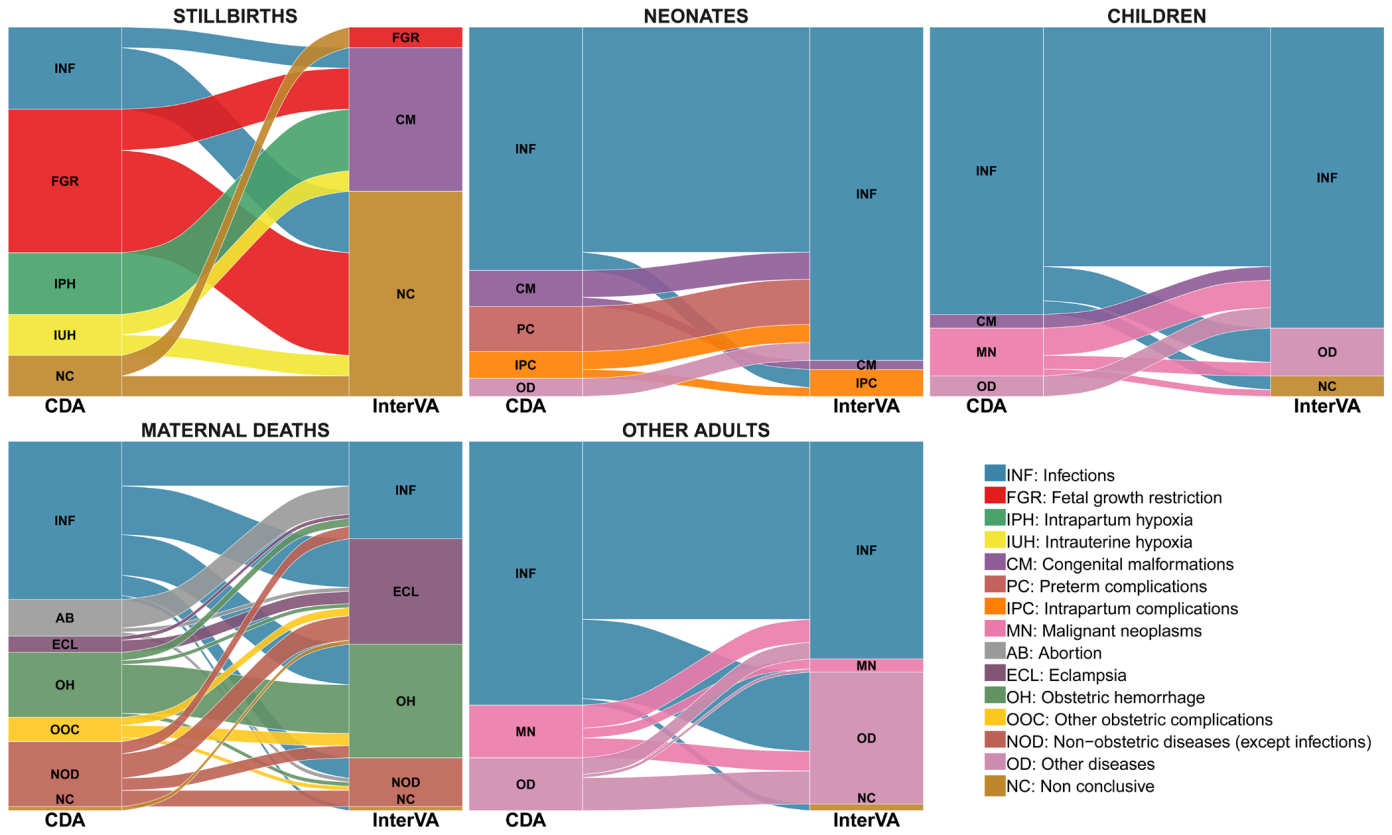
Alluvial diagrams of the differences in assignment of individual causes of death established by the Complete Diagnostic Autopsy (CDA) and InterVA (Interpreting Verbal Autopsy) model by study group. The stacked blocks represent the causes of death (CoDs) determined by the CDA (left) and by the InterVA model (right), and their size as proportional to the cause-specific mortality fractions (CSMFs). The branches between blocks represent differences in the composition of the CoDs between the CDA and the InterVA model, being their thickness proportional to the number of cases contained in both blocks connected by the branch. Each CoD is represented by a different color, which is the same in both diagnostic methods. The color of the branches is determined by the cause of actual death (CDA). The concordant cases between the CDA and the InterVA model are represented by branches connected to blocks of the same color. In contrast, misclassified cases are shown as branches connected to blocks of different color.

Table 2 shows the measures of overall concordance between the two methods corrected for chance by study group for all CoDs. The CCC ranged between -0.093 and 0.246, and Kappa statistic ranged from -0.030 to 0.232 (lowest in stillbirths and highest in other adults). Figure 1 presents the alluvial diagrams showing the differences in the assignment of individual CoD established in the two methods by study group.

**Table 2.** Measures of performance of the InterVA (Interpreting Verbal Autopsy) model compared to the complete diagnostic autopsy at individual-level prediction for all causes of death by study group.

Study group	CCC	Kappa	
Stillbirths	-0.093	-0.030	(poor)
Neonates	0.119	0.142	(slight)
Children	0.020	0.020	(slight)
Maternal deaths	0.179	0.159	(slight)
Other adults	0.246	0.232	(fair)

**CCC:** Chance-corrected concordance calculated from 500 Dirichlet draws

### Cause of death assignment of the model at the individual level among patients dying of infectious diseases

**Figure 2.**
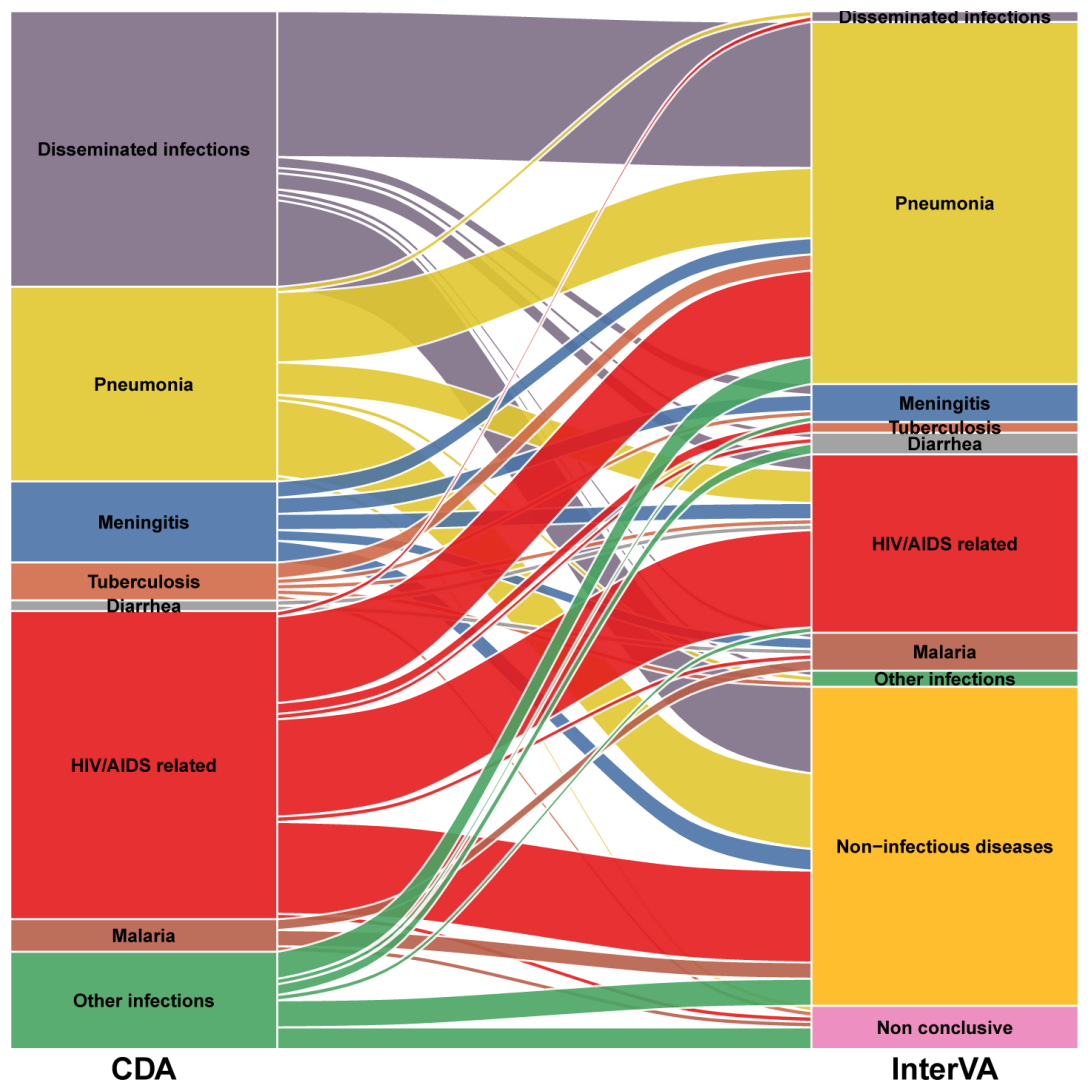
Alluvial diagrams of the differences in the individual cause of death as established by the complete diagnostic autopsy (CDA) and the InterVA (Interpreting Verbal Autopsy) model among patients who died of infectious diseases. The stacked blocks represent the causes of death (CoDs) determined by the CDA (left) and by the InterVA model (right), and their size as proportional to the cause-specific mortality fractions (CSMFs). The branches between blocks represent differences in the composition of the CoDs between the CDA and the InterVA model, being their thickness proportional to the number of cases contained in both blocks connected by the branch. Each CoD is represented by a different color, which is the same in both diagnostic methods. The color of the branches is determined by the cause of actual death (CDA). The concordant cases between the CDA and the InterVA model are represented by branches connected to blocks of the same color. In contrast, misclassification cases are shown as branches connected to blocks of different color. **Diseminated infections:** bacterial sepsis of the newborn (n=21), puerperal sepsis (n=6), streptococcal sepsis (n=5) and other sepsis (n=19) **HIV/AIDS related infections:** candidiasis (n=1), congenital viral diseases (n=1), cryptococcosis (n=11), cytomegaloviral disease (n=7), herpes simplex infection (n=1), miliary tuberculosis (n=20), salmonella infection (n=1), pneumocystosis (n=5), respiratory tuberculosis bacteriologically and histologically confirmed (n=2), toxoplasmosis (n=7) and tuberculous meningitis (n=1) **Other infections:** acute pericarditis (n=2), pyelonephritis (n=2), congenital viral diseases (n=2), chorioamnionitis (n=2), GBS infection (n=2), tetanus (n=1), peritonitis (n=3), rabies (n=3) and zygomycosis (n=1) **Non-infectious diseases (by the InterVA model):** congenital malformations (n=1), intrapartum complication (n=2), eclampsia (n=12), obstetric haemorrhage (n=10), non-obstetric diseases (n=5)and other diseases (n=29).

Table 3 shows the performance of the VA model in assigning CoD among cases who died of an infectious disease according to the CDA in all study groups by infection category. The sensitivity of the model in identifying an infectious disease as CoD was low for all infectious categories, being 0% for tuberculosis, diarrhea, disseminated and other infections. Figure 2 shows the alluvial diagram of the comparison of CoD assigned by both methods.

**Table 3.** Performance of the InterVA (Interpreting Verbal Autopsy) model at individual-level prediction among all cases who died of infectious diseases according to the complete diagnostic autopsy.

Cause of death (CDA)	n	Classification of cases				Sensitivity (%)	Specificity (%)	PPV (%)	NPV (%)
		TP	TN	FP	FN				
Disseminated infections	51	0	139	2	51	0	99	0	73
Pneumonia	36	13	102	54	23	36	65	19	82
Meningitis	15	3	173	4	12	20	98	43	94
Tuberculosis	7	0	183	2	7	0	99	0	96
Diarrhoea	2	0	186	4	2	0	98	0	99
HIV/AIDS related	57	18	120	15	39	32	89	55	75
Malaria	6	2	181	5	4	33	97	29	98
Other infections	18	0	171	3	18	0	98	0	90

**CDA:** complete diagnostic autopsy; **n**: number of cases; **TP:** true positives; **TN:** true negatives; **FP:** false positives; **FN:** false negatives; **PPV:** positive predictive value; **NPV:** negative predictive value; **N/A:** not applicable **Disseminated infections:** bacterial sepsis of newborn (n=21), puerperal sepsis (n=6), streptococcal sepsis (n=5) and other sepsis (n=19) **HIV/AIDS related infections:** candidiasis (n=1), congenital viral diseases (n=1), cryptococcosis (n=11), cytomegaloviral disease (n=7), herpes simplex infection (n=1), miliary tuberculosis (n=20), salmonella infections (n=1), pneumocystosis (n=5), pulmonary tuberculosis (n=2), toxoplasmosis (n=7) and tuberculous meningitis (n=1) **Other infections:** acute pericarditis (n=2), pyelonephritis (n=2), congenital viral diseases (n=2), chorioamnionitis (n=2), GBS infection (n=2), tetanus (n=1), peritonitis (n=3), rabies (n=3) and zygomycosis (n=1)

### Cause of death assignment of the model at the population-level, compared with the CDA

Table 4 shows the CSMFs estimated by the VA model and the CDA aggregated into broad categories of CoD. In stillbirths, the most frequent CoD assigned by the model was congenital malformation (39%); however, no case of congenital malformation was identified by the CDA. In addition, fetal growth restriction (FGR) was the most frequent CoD in stillbirths determined by the CDA (39%), but only one case was estimated as such by the model. Infectious diseases were responsible for 22% of stillbirths by the CDA, but no stillbirth was assigned to infectious diseases by the VA model. According to the VA model, no deaths were assigned to preterm complications in neonates, while these represented 12% of the neonatal deaths by the CDA. Among children, malignant neoplasms accounted for 13% of the deaths in the CDA, but no case was assigned to this CoD in the model. The model identified eclampsia as the second most prevalent cause of maternal mortality while only in four (4%) cases eclampsia was the cause of maternal death by the CDA. Complications of abortion was diagnosed in nine (10%) cases, none of them being identified by the VA method.

**Table 4.** Cause-Specific Mortality Fractions and associated measures of validation of the InterVA (Interpreting Verbal Autopsy) model compared with the complete diagnostic autopsy (CDA) at the population level.

Group	Cause of death	InterVA		CDA		CSMF Accuracy	
		n*	CSMF (%)	n	CSMF (%)	Uncorrected	Chance Corrected
Stillbirths	Infections	0	0.0	4	22.2	0.11	-1.00
	Fetal growth restriction	1	5.0	7	38.9		
	Intrapartum hypoxia	0	0.0	3	16.7		
	Intrauterine hypoxia	0	0.0	2	11.1		
	Congenital malformations	7	38.9	0	0.0		
	Non conclusive	10	56.1	2	11.1		
	*Overall*	*18*	*100.0*	*18*	*100.0*		
Neonates	Infections	37	91.0	27	65.9	0.73	0.50
	Congenital malformations	1	2.4	4	9.8		
	Preterm complications	0	0.0	5	12.2		
	Intrapartum complications	2	4.5	3	7.3		
	Other diseases	0	0.0	2	4.9		
	Non-conclusive	1	2.1	0	0.0		
	*Overall*	*41*	*100.0*	*41*	*100.0*		
Children	Infections	40	73.9	42	77.8	0.77	0.62
	Congenital malformations	0	0.0	2	3.7		
	Malignant neoplasms	0	0.0	7	13.0		
	Other diseases	7	13.7	3	5.6		
	Non-conclusive	7	12.5	0	0.0		
	*Overall*	*54*	*100.0*	*54*	*100.0*		
Maternal deaths	Infections *	22	24.1	39	42.9	0.59	-0.05
	Abortion	0	0.0	9	9.9		
	Eclampsia	25	27.5	4	4.4		
	Obstetric hemorrhage	26	28.1	16	17.6		
	Other obstetric complications	0	0.4	6	6.6		
	Non-obstetric diseases **	11	12.0	16	17.6		
	Non-conclusive	7	7.9	1	1.1		
	*Overall*	*91*	*100.0*	*91*	*100.0*		
Other adults	Infections	65	58.2	80	71.4	0.76	0.49
	Malignant neoplasms	4	3.7	16	14.3		
	Other diseases	33	29.8	16	14.3		
	Non-conclusive	9	8.4	0	0.0		
	*Overall*	*112*	*100.0*	*112*	*100.0*		

**CDA:** Complete diagnostic autopsy; n*: sum of cases estimated by InterVA model (in cause 1 or 2) weighted by their associated likelihood. The residual likelihoods count as non-conclusive case fractions; **n:** sum of cases established by the CDA; **CSMF:** cause-specific mortality fractions; CSMF Accuracy:measures the quality at the population level, quantifying how closely the estimated CSMF values approximate the truth; **Uncorrected:** median cause-specific mortality fractions accuracy across 500 Dirichlet draws. It ranges from zero to one; **Chance corrected:** Median Cause-Specific Mortality Fractions Accuracy for random allocation across 500 Dirichlet draws. A score of zero indicates predictive accuracy equal to random allocation. * Includes all infections, both obstetric and non-obstetric. ** Non-obstetric diseases do not include infections.

The model was less accurate in stillbirths (CSMFA of 0.11) than in the other groups (CSMFA ranging from 0.59 to 0.77). When corrected by chance, the accuracy of the model compared to the CDA was not better than that expected by chance in stillbirths (negative CCCSMFA), close to chance in maternal deaths (close to zero CCCSMFA) and better than that expected by chance but far from perfection in the other groups ( Table 4).

## Discussion

It is recognized that accurate information on what is causing deaths is essential to reduce mortality. In this study, we have assessed for the first time to our knowledge, the validity of a commonly used VA method in establishing the CoD compared with the gold standard (the CDA) in different age groups of patients dying at a tertiary-level hospital in Maputo, Mozambique. The agreement of the VA model was overall poor across all age groups and conditions, both at the individual and at the population level.

The two reference standards that have been used for validating computer-coded VA, i.e. physician-coded VA methods and health facility medical records, cannot be considered true gold standards. The comparison between computer-coded and physician-coded VA methods lacks an external reference or gold standard comparator ^11, 31^. On the other hand, although health facility-derived information is considered as an appropriate reference standard for VA validation ^27^, reports from both high and low-income countries indicate that this information frequently contains clinical errors ^4, 5^. It seems quite evident that if the main source of input to the VA tool is inaccurate, the output of the VA will not be precise either. Furthermore, if clinical errors are frequent even in well-equipped hospitals, it is expected that their frequency would be higher in VA data.

In this study, the performance of the VA model was overall poor in identifying CoDs in stillbirths. These findings disagree with those of a report from Pakistan using clinical data as reference standard, indicating that a physician-coded VA tool was valid to ascertain causes of stillbirths, specially congenital malformations ^32^. In neonates, the sensitivity of the model in identifying preterm complications as a CoD, was also very low (0%), which may be relevant for pre-term birth prevention programs. In contrast, the performance of the model in identifying infectious diseases as a cause of neonatal death had a high sensitivity, suggesting that it may be an adequate method to identify neonatal sepsis at the community. Among children, the sensitivity of the model was only high in detecting infectious diseases as a CoD but it did not identify deaths due congenital malformations and malignant neoplasms. In maternal deaths, the sensitivity of the model was high in assigning eclampsia as a cause of maternal mortality; however, the probability that a maternal death identified as eclampsia by the model was actually eclampsia was quite low. Although there were 26 maternal deaths assigned to eclampsia as the most probable CoD according to the VA model, only four were actually due to eclampsia (most misdiagnosed cases died of infectious diseases), suggesting a significant overestimation of eclampsia as a cause of maternal mortality by this method. This is in agreement with a previous report where a high frequency of false positive clinical diagnosis of eclampsia compared to the CDA was also found, being most of them deaths from infectious diseases ^4^. These findings are of relevance to eclampsia prevention programs, which may fail in reducing maternal mortality due to misdiagnosis. In adults, the sensitivity of the VA model was higher for infectious diseases compared to other CoD, but low in identifying malignant neoplasms as cause of mortality. According to these results, the model would underestimate malignant neoplasms as CoD in adults, which may be important for prevention programs of this condition in high mortality settings.

The performance of the model in identifying the specific infection CoD among patients who died of an infectious disease was overall low. The sensitivity of the model in identifying tuberculosis as a CoD was very low, which may be of public health relevance in high burden countries. Regarding malaria infection, the VA model and the CDA only agreed in two cases, while in the other four cases established by the CDA, the model assigned three of them to a non-infectious disease CoD and one as non-conclusive ( Figure 2). Lack of precision at the individual level in assigning malaria infection as a CoD may be important to target malaria control efforts in the community and increasing programme’s effectiveness.

The main use of the VA information is to determine cause-specific mortality and distribution of CoD at the population level ^33^; for this reason we also estimated the CSMF accuracy between the two methods. Both methods differed in the distribution of the proportion of the deaths assigned to several disease categories. When corrected by chance, the accuracy of the model in predicting in the population the CoD was poor, especially in stillbirths and maternal deaths and imperfect in the other groups.

A possible limitation of our study that might have influenced the predictions of the model, is that the indicators used to estimate the CoD by the VA model were extracted from medical records, since VAs were not done, which relates to the absence in the clinical records of some indicators of the model (43 indicators, 18% of the total). Nevertheless, most of these indicators (n=24) were secondary questions related to the duration of the event and therefore, not pertinent if the event did not occur. On the other hand, the most likely explanation for the lack of registration in the clinical record of the other 19 indicators (8%) is that they were not identified. The fact that the study is based in a large hospital might be seen as a limitation to extrapolate findings to deaths occurring in rural health-facilities or at home, since cause-composition of deaths in the community may be different to that of those occurring in a hospital. However, it is important to remember that this is a validation study and therefore, the objective was not that the deaths included were representative of those occurring in the community, but rather that the comparator of the VA was as true gold standard as possible. Thus, we needed a set of deaths, whose causes were established by the CDA, and therefore they had to occur in a hospital setting with autopsy facilities. On the other hand, to avoid that the cause-composition of deaths in that particular hospital and/or time-period affected the accuracy of the estimates of the VA, we created multiple test datasets with widely varying cause-compositions as it has been suggested ^28^.

As explained in the methods section, we used InterVA version 4 because version 5 was not available at the time of this analysis. Even if the estimated CoD might differ between the two InterVA versions, a change in the group of CoD would not be expected. Otherwise it would mean that the two versions provide different results, requiring a revision of all published information using the previous InterVA model.

The post-2015 Development Agenda expects that high burden countries should have reliable information on number and CoD to reduce their main health problems ^34^. However, this goal cannot continue to rely on imprecise measurement tools. The main shortcoming to achieve the SDGs is the imprecision of the currently used methods to establish CoD. These findings highlight the need of improving the quality and performance of current VA techniques by developing more precise tools for CoD ascertainment.

In conclusion, the “data revolution” of the post-2015 Development Agenda expects that high burden countries should have reliable information on number and causes of death in order to reduce main health problems through evidence-based decision-making, and target and monitor health programs ^29^. However, this goal cannot continue to rely on imprecise measurement tools. The main shortcoming to achieve the SDGs, is not the scarce availability of physicians to carry out death certificates or VA codification, nor the solution is the available automated methods created to overcome some of the physician-coded VA limitations, but rather the imprecision of these methods to reliably establish causes of death. The findings of this study should serve to highlight the need to implement autopsy methods where they may be feasible, but even more importantly to improve the quality and performance of current VA techniques and to develop more precise CoD ascertainment tools.

### Consent

Written informed consent for publication of the patients’ details and/or their images was obtained from the parents/guardian/relative of the patient.

## Data availability

### Underlying data

Study data cannot be shared in a public domain due to their sensitive nature and, being such as small sample, especially for some age-specific causes of death, it would be relatively easy to identify study individuals even if anonymized. However, deidentified data will be made available from the corresponding author on reasonable request. Requesters will be required to sign a letter of agreement detailing the mechanisms by which the data will be kept secure and access restricted to their study team. The agreements will also state that the recipient will not share the data with anyone outside of their research team. 

### Extended data

Open Science Framework: Limitations to current methods to estimate cause of death: a validation study of a verbal autopsy model.

https://doi.org/10.17605/OSF.IO/UMJV2 ^25^

This project contains the following extended data:

-     Clinical_questionnaire.pdf (Clinical and epidemiological data collection questionnaire)
-     VA_validation_extended_data.pdf (PDF containing supplementary figures and tables)
        Figure S1. Outline of statistical methods
        Table S1. Description of metrics
        Table S2. Study group and number of causes of death and their associated likelihoods as established by the InterVA method
        Table S3. Number of cases and mean likelihood agreement between the InterVA’s predicted cause of death and that established by the CDAby study group

Data are available under the terms of the Creative Commons Zero “No rights reserved” data waiver (CC0 1.0 Public domain dedication).

## References

[1] The World Bank: World development report 1993: investing in health. New York, Oxford University Press,1993. Reference Source

[2] Mikkelsen L Phillips DE AbouZahr C: A global assessment of civil registration and vital statistics systems: monitoring data quality and progress. *Lancet Lond Engl.* 2015;386(10001):1395–1406. 10.1016/S0140-6736(15)60171-4 25971218

[3] Shojania KG Burton EC McDonald KM: Changes in rates of autopsy-detected diagnostic errors over time: a systematic review. *JAMA.* 2003;289(21):2849–2856. 10.1001/jama.289.21.2849 12783916

[4] Ordi J Ismail MR Carrilho C: Clinico-pathological discrepancies in the diagnosis of causes of maternal death in sub-Saharan Africa: retrospective analysis. *PLoS Med.* 2009;6(2):e1000036. 10.1371/journal.pmed.1000036 19243215 PMC2646780

[5] Taylor TE Fu WJ Carr RA: Differentiating the pathologies of cerebral malaria by postmortem parasite counts. *Nat Med.* 2004;10(2):143–145. 10.1038/nm986 14745442

[6] Quigley MA Schellenberg JRA Snow RW: Algorithms for verbal autopsies: a validation study in Kenyan children. *Bull World Health Organ.* 1996;74(2):147–154. 8706229 PMC2486900

[7] World Health Organization and others: Verbal autopsy standards: ascertaining and attributing cause of death. Geneva: World Health Organization,1993. Reference Source

[8] Leitao J Desai N Aleksandrowicz L: Comparison of physician-certified verbal autopsy with computer-coded verbal autopsy for cause of death assignment in hospitalized patients in low-and middle-income countries: systematic review. *BMC Med.* 2014;12:22. 10.1186/1741-7015-12-22 24495312 PMC3912516

[9] Byass P Kahn K Fottrell E: Moving from data on deaths to public health policy in Agincourt, South Africa: approaches to analysing and understanding verbal autopsy findings. *PLoS Med.* 2010;7(8):e1000325. 10.1371/journal.pmed.1000325 20808956 PMC2923087

[10] Bauni E Ndila C Mochamah G: Validating physician-certified verbal autopsy and probabilistic modeling (InterVA) approaches to verbal autopsy interpretation using hospital causes of adult deaths. *Popul Health Metr.* 2011;9:49. 10.1186/1478-7954-9-49 21819603 PMC3160942

[11] Tensou B Araya T Telake DS: Evaluating the InterVA Model for Determining AIDS Mortality From Verbal Autopsies in the Adult Population of Addis Ababa. *Trop Med Int Health.* 2010;15(5):547–53. 10.1111/j.1365-3156.2010.02484.x 20214760 PMC3901008

[12] Ministerio da Saude - MISAU/Moçambique, Instituto Nacional de Estatística - INE/Moçambique, ICF International. *Moçambique Inquérito Demográfico e de Saúde 2011*. Calverton, Maryland, USA: MISA/Moçambique, INE/Moçambique and ICF International,2013. Reference Source

[13] UNAIDS: Ending AIDS. Progress towards the 90-90-90 targets. Global AIDS update 2017,2017. Reference Source

[14] Castillo P Martínez MJ Ussene E: Validity of a Minimally Invasive Autopsy for Cause of Death Determination in Adults in Mozambique: An Observational Study. *PLoS Med.* 2016;13(11):e1002171. 10.1371/journal.pmed.1002171 27875530 PMC5119723

[15] Menendez C Castillo P Martínez MJ: Validity of a Minimally Invasive Autopsy for Cause of Death Determination in Stillborn Babies and Neonates in Mozambique: An observational study. *PLoS Med.* 2017;14(6):e1002318. 10.1371/journal.pmed.1002318 28632735 PMC5478138

[16] Bassat Q Castillo P Martínez MJ: Validity of a minimally invasive autopsy tool for cause of death determination in pediatric deaths in Mozambique: An observational study. *PLoS Med.* 2017;14(6):e1002317. 10.1371/journal.pmed.1002317 28632739 PMC5478091

[17] Castillo P Hurtado JC Martínez MJ: Validity of a minimally invasive autopsy for cause of death determination in maternal deaths in Mozambique: An observational study. *PLoS Med.* 2017;14(11):e1002431. 10.1371/journal.pmed.1002431 29117196 PMC5695595

[18] Castillo P Ussene E Ismail MR: Pathological methods applied to the investigation of causes of death in developing countries: minimally invasive autopsy approach. *PLoS One.* 2015;10(6):e0132057. 10.1371/journal.pone.0132057 26126191 PMC4488344

[19] World Health Organization: ICD-10 Volume 2 Instruction Manual.2016. Reference Source

[20] Leitao J Chandramohan D Byass P: Revising the WHO verbal autopsy instrument to facilitate routine cause-of-death monitoring. *Glob Health Action.* 2013;6:21518. 10.3402/gha.v6i0.21518 24041439 PMC3774013

[21] Byass P Herbst K Fottrell E: Comparing Verbal Autopsy Cause of Death Findings as Determined by Physician Coding and Probabilistic Modelling: A Public Health Analysis of 54 000 Deaths in Africa and Asia. *J Glob Health.* 2015;5(1):010402. 25734004 10.7189/jogh.05.010402 PMC4337147

[22] Fottrell E Byass P: Verbal Autopsy: Methods in Transition. *Epidemiol Rev.* 2010;32:38–55. 10.1093/epirev/mxq003 20203105

[23] Byass P Chandramohan D Clark SJ: Strengthening Standardised Interpretation of Verbal Autopsy Data: The New InterVA-4 Tool. *Glob Health Action.* 2012;5:1–8. 10.3402/gha.v5i0.19281 22944365 PMC3433652

[24] Fottrell E Byass P Ouedraogo TW: Revealing the Burden of Maternal Mortality: A Probabilistic Model for Determining Pregnancy-Related Causes of Death from Verbal Autopsies. *Popul Health Metr.* 2007;5:1. 10.1186/1478-7954-5-1 17288607 PMC1802065

[25] Quintó L: Limitations to current methods to estimate cause of death: a validation study of a verbal autopsy model.2020. 10.17605/OSF.IO/UMJV2 PMC7590499 33145479

[26] Li ZR McCormick TH Clark SJ: InterVA4: An R package to analyze verbal autopsy data. Center for Statistics and the Social Sciences, University of Washington: Vienna, Austria: R Foundation for Statistical Computing. [1044],2014. Reference Source

[27] Murray CJ Lopez AD Black R: Population Health Metrics Research Consortium gold standard verbal autopsy validation study: design, implementation, and development of analysis datasets. *Popul Health Metr.* 2011;9:27. 10.1186/1478-7954-9-27 21816095 PMC3160920

[28] Murray CJ Lozano R Flaxman AD: Robust metrics for assessing the performance of different verbal autopsy cause assignment methods in validation studies. *Popul Health Metr.* 2011;9:28. 10.1186/1478-7954-9-28 21816106 PMC3160921

[29] Desai N Aleksandrowicz L Miasnikof P: Performance of four computer-coded verbal autopsy methods for cause of death assignment compared with physician coding on 24,000 deaths in low-and middle-income countries. *BMC Med.* 2014;12:20. 10.1186/1741-7015-12-20 24495855 PMC3912488

[30] Flaxman AD Serina PT Hernandez B: Measuring causes of death in populations: a new metric that corrects cause-specific mortality fractions for chance. *Popul Health Metr.* 2015;13:28. 10.1186/s12963-015-0061-1 26464564 PMC4603634

[31] Oti SO Kyobutungi C: Verbal autopsy interpretation: a comparative analysis of the InterVA model versus physician review in determining causes of death in the Nairobi DSS. *Popul Health Metr.* 2010;8:21. 10.1186/1478-7954-8-21 20587026 PMC2902422

[32] Nausheen S Soofi SB Sadiq K: Validation of verbal autopsy tool for ascertaining the causes of stillbirth. *PLoS One.* 2013;8(10):e76933. 10.1371/journal.pone.0076933 24130814 PMC3793932

[33] Byass P Fottrell E Huong DL: Refining a probabilistic model for interpreting verbal autopsy data. *Scand J Public Health.* 2006;34(1):26–31. 10.1080/14034940510032202 16449041 PMC2833983

[34] High Level Panel: The Post 2015 Development Agenda.2018. Reference Source

