# Supplementary material for: Limitations to current methods to estimate cause of death: a validation study of a verbal autopsy model
Source: Gates Open Res. 2021 May 5;4:55. Originally published 2020 May 28. [Version 3] doi: 10.12688/gatesopenres.13132.3 (PMC7590499; doi:10.12688/gatesopenres.13132.3)
Supplement: Peer review history

# Peer review: Reviewer response for version 1

**DOI:** 10.21956/gatesopenres.14319.r29704

**Published:** 2020-10-23

**Authors:** Noriah Maraba

**Affiliations:** 1 The Aurum Institute, Johannesburg, South Africa

The manuscript is scientifically sound, well written and contributes to body of literature on the validity of VA in assigning CoDs. There are just few suggestions that I think will assist with better organization of the manuscript. There are a few areas where there are grammatical errors that need to be addressed.

 There is inconsistence in the way InterVA model is being referred to in the manuscript as it is sometimes called the VA model and in other places model. I would recommend that the authors refer to the model by its name and not as 'VA model' as there are a number of other computer coded models for VA.

 The authors indicate the number of CAD that were completed in the methods section and that number could go into the results section. I recommend the same for the paragraph describing the population of decedents, which can also go into results section.

 The authors could start off the results section with a paragraph describing the population of decedents according to age groups. It would also be good to include a paragraph or two just describing the top CoDs assigned by the two methods for each study group before comparing them.

 The heading of the second paragraph in the results could be changed to ‘’Agreement/comparison of COD at individual level between the two methods’’ as its describing agreement and not just assignment.

 The authors are not providing source data because of issues of confidentiality.

Is the work clearly and accurately presented and does it cite the current literature?

Yes

If applicable, is the statistical analysis and its interpretation appropriate?

Yes

Are all the source data underlying the results available to ensure full reproducibility?

No

Is the study design appropriate and is the work technically sound?

Yes

Are the conclusions drawn adequately supported by the results?

Yes

Are sufficient details of methods and analysis provided to allow replication by others?

Yes

Reviewer Expertise:

NA

I confirm that I have read this submission and believe that I have an appropriate level of expertise to confirm that it is of an acceptable scientific standard.

# Peer review: Reviewer response for version 1

**DOI:** 10.21956/gatesopenres.14319.r29737

**Published:** 2020-10-21

**Authors:** Clara Calvert

**Affiliations:** 1 Department of Population Health, London School of Hygiene & Tropical Medicine, London, UK

**General assessment:**

 Thank you for the opportunity to review this very clearly written article. As the first study to compare cause of death assignment from Verbal Autopsy (VA) based on automated methods to cause of death assignment based on complete diagnostic autopsy, this is a very useful contribution to the field. Overall, the authors make a coherent argument, though I have a few suggestions on how certain aspects could be clarified or improved.

**Introduction:**

 Generally, this is very clearly written and provides a clear rationale for this research. I have just minor comments, as follows:

1. The final sentence of the first paragraph is slightly confusing, implying that certain conditions need to be tracked using clinical records and VAs, where I think it is all conditions in certain settings that require use of clinical records and/or VAs. I would suggest amending the final sentence of first paragraph to state that cause of death (CoD) distributions in many low-income settings rely on estimates based on clinical records.
2. You note that clinical errors mean that VA remains the most practical approach for estimating CoD at the beginning of the second paragraph; however, I think one of the main reasons for using VA is to capture deaths that occur in the community.
3. The definition of a verbal autopsy in this paper specifies that the interview is interpreted by a physician, but I would argue that this is one method to interpret the data from the VA and the other is automated methods. It would be good to clarify this.
4. When describing the diagnostic accuracy of VA interpreted using physician review in the second paragraph, you provide a reference to the study by Quigley *et al.*; this study looks only at children so it would be good to provide further reference to the study which showed poor diagnostic accuracy for meningitis in all age groups.

 

**Methods:**

1. It would be helpful to provide an indication of which deaths are likely to have a complete diagnostic autopsy (CDA) requested and possibly a rough idea of how many deaths receive a CDA request. My suspicion – which may be completely unfounded – is that these deaths may represent particularly tricky deaths to assign cause of death given that a CDA was requested by the clinician. This would have important implications on the interpretation of the results.
2. You write that InterVA-4 has the advantage of being “standardized to interpretation” – can you clarify what this means?
3. Could you add a citation for the R package for InterVA-4 in the methods?
4. You mention that the data were extracted from the clinical record, and it would be helpful to have some more clarification on this tool – initially I had assumed that this was a medical record in line with what was routinely used in the hospital; however, this looks much more like a questionnaire that was designed specifically as part of the project – it would be useful to know if this was the case and, if so, who completed this questionnaire.  
5. In the validation of the model, you write that you generated 500 cause compositions across “each study group” – which study groups are you referring to? Do you mean by age and separating out maternal deaths? It would be good to include a statement that clarifies that “All analyses were undertaken by the following groups: stillbirth, neonates, children, pregnant and postpartum women and other adults.”
6. More generally, I felt that the “validation of the model” was lacking in detail. It would be helpful to make the rationale for generating 500 cause compositions clear. You also present sensitivity, specificity, PPV and NPV in the results but do not mention the calculation of these in the methods; at the very least you should mention that you calculate these and use the CDA as the gold standard.   

**Results:**

1. As a very minor point, it would be better to use the term InterVA-4 throughout the paper, rather than referring to it as the VA model.
2. Change reference to “tumours” in text to “malignant neoplasms” to match terminology used in Table 1.
3. The tables and figures are very clear; I particularly like the alluvial diagrams which are a very nice way to present these findings. Note that there is a small typo in the title of Figure one: change “misclassification” to “misclassified”.

**Discussion:**

 A possible additional point to make is that one of the key drivers of the outputs from the InterVA-4 algorithm is the Symptom-Cause Information (SCI) which was initially derived from a group of physicians; information that is generated from studies like this has the potential to inform future iterations of the SCI to improve the performance of the automated methods (and newer versions of the algorithms are increasing opportunities for studies to specify their own SCI).

Is the work clearly and accurately presented and does it cite the current literature?

Yes

If applicable, is the statistical analysis and its interpretation appropriate?

Yes

Are all the source data underlying the results available to ensure full reproducibility?

Partly

Is the study design appropriate and is the work technically sound?

Yes

Are the conclusions drawn adequately supported by the results?

Yes

Are sufficient details of methods and analysis provided to allow replication by others?

Partly

Reviewer Expertise:

maternal health; verbal autopsy; HIV

I confirm that I have read this submission and believe that I have an appropriate level of expertise to confirm that it is of an acceptable scientific standard, however I have significant reservations, as outlined above.

# Peer review: Reviewer response for version 1

**DOI:** 10.21956/gatesopenres.14319.r29256

**Published:** 2020-08-19

**Authors:** Abdul Wahab

**Affiliations:** 1 Faculty of Medicine, Public Health and Nursing, Universitas Gadjah Mada, Yogyakarta, Indonesia; 2 Center for Reproductive Health, Universitas Gadjah Mada, Yogyakarta, Indonesia

This article is very interesting as a scientific work that explains the validation of the verbal autopsy model. The research problems have been described quite clearly and the research methods were presented in detail and were accurate. The results are presented clearly and perfectly with proper and in-depth discussion using sufficient references. The conclusion drawn was adequately supported by the results and discussion. But, perhaps there should be provided recommendations for researchers and practitioners regarding the use of verbal autopsy data in determining the cause of death.

Is the work clearly and accurately presented and does it cite the current literature?

Yes

If applicable, is the statistical analysis and its interpretation appropriate?

I cannot comment. A qualified statistician is required.

Are all the source data underlying the results available to ensure full reproducibility?

Partly

Is the study design appropriate and is the work technically sound?

Yes

Are the conclusions drawn adequately supported by the results?

Yes

Are sufficient details of methods and analysis provided to allow replication by others?

Yes

Reviewer Expertise:

Public health, health demography, epidemiology, research methodology, adolescent reproductive health, maternal and child health.

I confirm that I have read this submission and believe that I have an appropriate level of expertise to confirm that it is of an acceptable scientific standard.
